# Role of noninvasive ocular imaging as a biomarker in peripheral artery disease (PAD): A systematic review

**DOI:** 10.1177/1358863X231210866

**Published:** 2023-12-06

**Authors:** Mallika Prem Senthil, Chroran Kurban, Ngoc Thuy Nguyen, Anh-Phuong Nguyen, Ranjay Chakraborty, Christopher Delaney, Robyn Clark, Saumya Anand, Heena Bhardwaj

**Affiliations:** 1 College of Nursing and Health Sciences, Caring Futures Institute, Flinders University, Adelaide, SA, Australia; 2 Department of Vascular and Endovascular Surgery, Flinders Medical Centre, Adelaide, SA, Australia

**Keywords:** fundus photography, ocular biomarker, optical coherence tomography angiography (OCTA), peripheral artery disease (PAD), retinal imaging

## Abstract

This study aimed to review the current literature exploring the utility of noninvasive ocular imaging for the diagnosis of peripheral artery disease (PAD). Our search was conducted in early April 2022 and included the databases Medline, Scopus, Embase, Cochrane, and others. Five articles were included in the final review. Of the five studies that used ocular imaging in PAD, two studies used retinal color fundus photography, one used optical coherence tomography (OCT), and two used optical coherence tomography angiography (OCTA) to assess the ocular changes in PAD. PAD was associated with both structural and functional changes in the retina. Structural alterations around the optic disc and temporal retinal vascular arcades were seen in color fundus photography of patients with PAD compared to healthy individuals. The presence of retinal hemorrhages, exudates, and microaneurysms in color fundus photography was associated with an increased future risk of PAD, especially the severe form of the disease. The retinal nerve fiber layer (RNFL) was significantly thinner in the nasal quadrant in patients with PAD compared to age-matched healthy individuals in OCT. Similarly, the choroidal thickness in the subfoveal region was significantly thinner in patients with PAD compared to controls. Patients with PAD also had a significant reduction in the retinal and choroidal circulation in OCTA compared to healthy controls. As PAD causes thinning and ischemic changes in retinal vessels, examination of the retinal vessels using retinal imaging techniques can provide useful information about early microvascular damage in PAD. Ocular imaging could potentially serve as a biomarker for PAD. **PROSPERO ID: CRD42022310637**

## Introduction

Peripheral artery disease (PAD) is a common vascular condition that is associated with atherosclerosis and reduced blood flow to the limbs. It affects 8–10 million people in the US and over 200 million people worldwide.^1–3^ PAD is a multifactorial disease and is associated with several risk factors such as diabetes, smoking, hypertension, hypercholesterolemia, and ischemic heart disease.^4–7^ It causes structural and functional changes in the limbs, leading to disability, reduced quality of life, high mortality, and substantial economic burden.^8–10^ Patients with PAD have a three to six times increased risk of cardiovascular mortality (myocardial infarction and stroke) compared to those without PAD^11^ and patients with severe symptomatic large-vessel PAD have a 25% chance of death by vascular causes within 12 months.^12^ The vasculature of the eye and the heart share several common characteristics and because the blood vessels of the eye are easily amenable to examination, they can to some extent serve as a window to the heart.^13^ Ocular signs such as retinal arteriovenous nipping, narrowing of the arteries, vasodilation of the retinal veins, and tortuosity of the retinal vessels are signs of cardiovascular risk.^14^ Risk factors for cardiovascular diseases such as diabetes, hypertension, hyperlipidemia, smoking, and obesity are also the risk factors for ocular diseases such as central retinal artery and vein occlusion, branch retinal artery and vein occlusion, age-related macular degeneration, and glaucoma.^15–18^ The retina is a unique site where microcirculation can be imaged directly. Different methods are available to examine the ocular structures and blood flow of the eye. Doppler color imaging is generally used to visualize extraocular blood flow and is mostly indicated in conditions such as central retinal artery occlusion and ophthalmic artery occlusion.^19^ Intraocular blood flow is visualized using fundus fluorescein angiography (FFA), indocyanine green (ICG) angiography, and color fundus photography. Optical coherence tomography (OCT) and optical coherence tomography angiography (OCTA) provide a high-resolution cross-sectional image of the retina and retinal and choroidal blood vessels, respectively. FFA and ICG are invasive procedures that require the use of dye to visualize the retinal and choroidal blood vessels.

Color fundus photography, OCT, and OCTA can enable early detection of retinal microvascular changes in patients with systemic conditions such as cardiovascular disease and PAD. Color fundus photography is a quick and simple noninvasive imaging technique but has several limitations. It is a two-dimensional imaging modality with low resolution and does not provide information about the microscopic changes in the retina that correspond to early stages of the disease (Table 1). On the other hand, OCT and OCTA are three-dimensional imaging modalities that have higher resolution than retinal color fundus photography. The main difference between OCT and OCTA is that OCT images the anatomical structure, whereas OCTA images the vascular structure. OCTA can provide information about both the structural and the blood flow changes in tandem, and is therefore considered a superior modality. OCTA imaging also has different scan types that can provide information about the different layers of retinal vessels. For example, the 3 × 3 mm scan with smaller field of view provides information about the small-caliber retinal vessels that are usually affected first in conditions such as PAD, and the 6 × 6 mm and the 8 × 8 mm scans have a wider field of view and provide information about the larger retinal arterioles and venules.

**Table 1.** Key components of retinal imaging techniques used in peripheral retinal disease.

Retinal imaging	Key components	Example images
Color fundus photography	• It is a two-dimensional imaging technique. • It uses a specialized camera to photograph the back of the eye called the fundus. • The fundus camera is a specialised low power microscope with an attached camera designed to photograph the interior of the eye including the retina, retinal blood vessels, optic disc, macula, and the posterior pole. • The eyes are well-dilated before the photography as it allows the camera to capture a greater area of the fundus and have a clearer view of the interior of the eye.	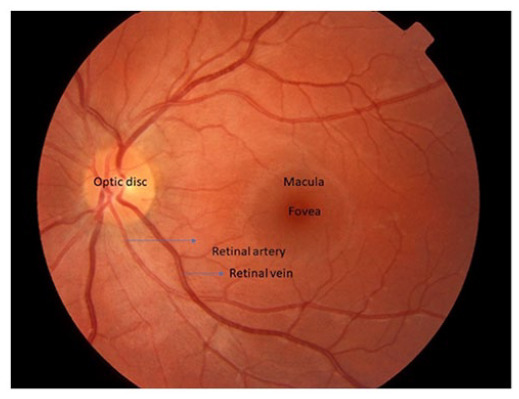
Optical coherence tomography (OCT)	• It is a new three-dimensional technique. • Enables high-resolution cross-sectional imaging of the retina, retinal nerve fiber layer and optic nerve head. • It uses a light source in the near-infrared spectrum. • The waves are directed to the tissue under examination where the waves reflect off the tissue structure. • It measures the time delay and amplitude of backscattered light using an interferometry to reconstruct the depth profile of the sample at the selected location. • A scanning OCT beam allows for acquisition of cross-sectional images of the tissue structure instantaneously. • Various OCTs have axial resolution of 20-5 μm in the tissue.	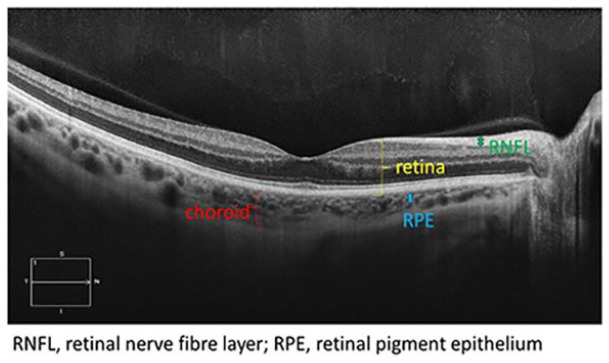
Optical coherence tomography angiography (OCTA)	• It is a three-dimensional technique that simultaneously gives structural and blood flow data. • It offers a highly accurate picture of the retinal vasculature and allows for the detection of minute microvascular anomalies. • It employs motion contrast imaging to high-resolution volumetric blood flow information generating angiographic images in a matter of seconds. • Unlike OCT, OCTA analyzes both the reflected light and the temporal variations of the OCT signal. • It compares the differences in the backscattered OCT signal intensity between sequential OCT b-scans taken at precisely the same cross-section to construct a map of blood flow. • It creates a map of blood flow by comparing the variations in the backscattered OCT signal intensity between successive OCT b-scans obtained at the same cross section.	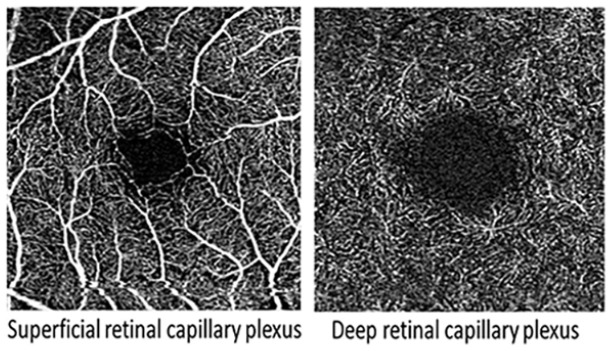

Note – images are in color online.

PAD is often asymptomatic leading to underdiagnosis and undertreatment.^12^ There is a lack of a noninvasive, reliable, and objective biomarker for screening of PAD.^20^ The most commonly used test for screening and diagnosing PAD is the ankle–brachial index (ABI). ABI values of 0.41–0.90 indicate mild to moderate PAD, and values less than 0.40 indicate severe PAD.^21^ However, as identified in a recent systematic review, the reliability of ABI for the diagnosis of PAD remains uncertain.^22^ Several inflammatory and genetic biomarkers have also been used for screening of PAD; however, these biomarkers have several disadvantages, such as being invasive, expensive, time consuming, and associated with complications.^23^ Ocular imaging is a noninvasive diagnostic method that enables qualitative and quantitative evaluation of the changes in the optic nerve head, nerve fiber layer, and macula, retinal and choroidal perfusion. These ocular biomarkers have been shown to be associated with many neurodegenerative and cardiovascular disorders.^24,25^ OCTA is a new noninvasive imaging technique that employs motion contrast imaging to high-resolution volumetric blood flow information for generating quick, high-resolution angiographic images of the retina and the choroid.^26^ Recent research has demonstrated that the OCTA can be used to evaluate the early microvascular damage caused by diabetes and may help identify diabetics who are more likely to develop diabetic retinopathy.^27^ PAD is associated with reduced blood flow to the retina and choroid,^28^ and therefore evaluation of the retinal and choroidal circulation using retinal imaging can provide valuable information about early microvascular damage in PAD.

This systematic review was undertaken to look at the current literature on the utility of ocular imaging biomarkers in the diagnosis of PAD.

## Methods

**Figure 1.**
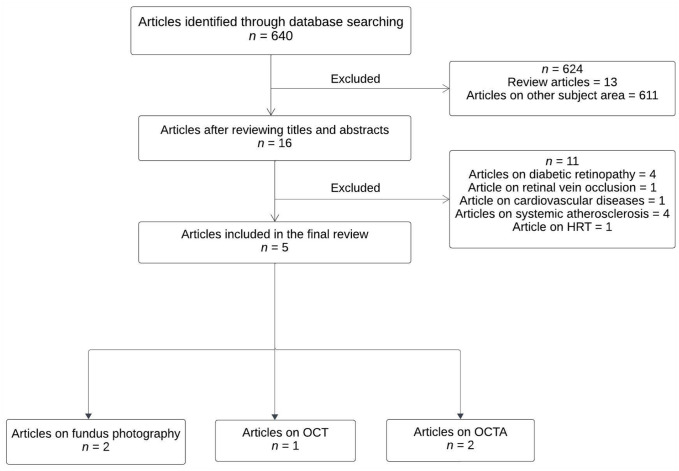
Flow chart showing the process of the systematic review. HRT, Heidelberg Retinal Tomography; OCT, Optical coherence tomography; OCTA, OCT angiography.

The Preferred Reporting Items for Systematic reviews and Meta-Analyses (PRISMA) standards were followed for conducting this systematic review. This review was registered with the International Prospective Register of Systematic Reviews (PROSPERO) (ID number CRD42022310637). The literature search was done using the following databases: Medline, Embase, Scopus, Cochrane, Latin Ameri-can and Caribbean Health Sciences Literature (LILACS), International Standard Randomised Controlled Trial Number (ISRCTN) registry, and International Clinical Trials Registry Platform (ICTRP). The search was performed on 15 April 2022 and was not limited to any preceding dates. We used the following search terms for our study: ‘*peripheral arterial disease*’, ‘*lower extremity arterial disease*’, ‘*hardening of the arteries*’, ‘*peripheral limb obstruction*’, ‘*lower extremity gangrene*’, ‘*retinal imaging*’, ‘*color fundus photography*’, ‘*optical coherence tomography*’, and ‘*optical coherence tomography angiography*’ (online supplementary material 1). Only original studies published on humans were included in the search. We included studies done on patients with any form of PAD. Studies with an unmatched design, such as case reports and reviews were excluded. The primary outcome was retinal and choroidal changes in PAD using noninvasive ocular imaging techniques. The search strategy and data abstraction were carried out by the authors HB, NN, APN, and CK and any disagreement was resolved by discussion. Figure 1 shows the steps involved in the systematic review process. The included articles were assessed for risk of bias using the National Institutes of Health Quality Assessment Tool for observational cohort, case–control, cross-sectional studies.

## Results

A total of 640 articles were identified from the initial search, from which 624 were excluded after reviewing their titles and abstracts. After comparing the remaining 16 articles to the selection criteria, 11 more items were removed. Our final review included five articles that explored the utility of ocular imaging biomarkers for the diagnosis of PAD (Table 2). The retrieved articles were categorized into three groups: (1) studies that used retinal color fundus photography; (2) studies that used optical coherence tomography (OCT); and (3) studies that used OCTA in PAD. Of the five studies that were included in this review, two studies used retinal color fundus photography, two used OCTA, and one used OCT as the imaging modality. The quality assessment of the included articles using the National Institutes of Health Quality Assessment Tool is shown in online supplementary material 2.

**Table 2.** Demographic details of studies included in the systematic review.

Year	Authors	Country	Study design	Sample size	Population	Retinal imaging	Results
2020	Yang et al.^29^	USA	Prospective study	PAD = 303 Controls = 9068	PAD and controls	CFP	Participants with retinal hemorrhages, exudates, and microaneurysms had a three to seven times higher risk of PAD compared to their counterparts without these retinopathy findings.
2021	Sun et al.^31^	Australia	Cross-sectional study	Cardiovascular patients = 247	Patients with cardiovascular disease	OCTA	Increasing age, hypertension, dyslipidemia, diabetes, chronic kidney disease, and PAD were associated with reduction in density of retinal vessels, vessel perfusion, average vessel length, and/or junction density in 3 × 3 mm OCTA.
2021	Soydan et al.^30^	Turkey	Case–control study	PAD = 35 Controls = 32	PAD and controls	OCT	Patients with PAD had a significant reduction in the retinal nerve fiber layer thickness in the nasal quadrant and subfoveal choroidal thickness compared to healthy controls.
2021	Wintergerst et al.^32^	Germany	Case–control study	PAD = 52 Controls = 23	PAD and controls	OCTA	Patients with PAD had a significant reduction in retinal and choroidal perfusion compared to healthy controls.
2022	Mueller et al.^20^	Germany	Case–control study	PAD = 135 Controls = 34	PAD	CFP	Alterations around the optic disc and retinal temporal vascular arcades were used to differentiate patients with PAD from healthy controls.

CFP, color fundus photography; OCT, optical coherence tomography; OCTA, optical coherence tomography angiography; PAD, peripheral artery disease.

### Retinal color fundus photography (CFP)

Two studies used retinal color fundus photography to evaluate the retinal changes in PAD. Mueller et al. used a deep learning architecture to process retinal color fundus photography images to compare different anatomical structures in patients with PAD (*n* = 135; mean age, 67.94 ± 9.47 years) and healthy controls (*n* = 34; mean age, 73 years), and reported that alterations around the optic disc and the temporal vascular arcades can be used to differentiate between patients with PAD and healthy controls.^20^ Yang et al. in their bi-racial community-based cohort (*n* = 303; mean age, 59.5 years) study showed that measures indicative of retinopathy, such as retinal hemorrhages, hard exudates, and microaneurysms, were independently and strongly associated with an increased future risk of PAD.^29^ Furthermore, they demonstrated that in subjects with underlying diabetes, retinopathy measurements were significantly associated with PAD.

### Optical coherence tomography (OCT)

There was one study that investigated the retinal vascular changes in PAD using OCT. Soydan et al., in their study, evaluated the retinal thickness, choroidal thickness (temporal, nasal, and subfoveal), retinal nerve fiber layer (RNFL) thickness (global, superior-temporal, superior-nasal, temporal, nasal, inferior-nasal, and inferior-temporal), and retinal artery and vein diameters in patients with PAD (*n* = 35; mean age, 59.4 ± 11.9 years) and healthy controls (*n* = 32; mean age, 55.5 ± 5.3 years), and showed that the subfoveal choroidal thickness was significantly lower in the PAD group (*p* = 0.03) compared to healthy individuals.^30^ Similarly, the RNFL thickness was significantly lower in the PAD group (*p* = 0.03) compared to healthy controls. However, no statistically significant difference was found between groups in arterial and vein diameters (*p* = 0.68), respectively.

### Optical coherence tomography angiography (OCTA)

In two studies, OCTA was used to detect the retinal and choroidal changes in patients with PAD and healthy controls. Sun et al. using a ZEISS CIRRUS HD-OCT model 5000 (Jena, Germany) studied the impact of cardiometabolic factors such as diabetes, hypertension, dyslipidemia, coronary artery disease, heart failure, chronic kidney disease, obesity, obstructive sleep apnea, and PAD on retinal vasculature. They used 3 × 3, 6 × 6, and 8 × 8 mm OCTA scans to quantify vascular parameters of the superficial capillary plexus layer in 247 patients with cardiovascular disease.^31^ They showed that changes in vessel density, vessel perfusion, average vessel length, and/or junction density were linked with ageing, hypertension, dyslipidemia, diabetes, chronic renal disease, coronary artery disease, and PAD in 3 × 3 mm OCTA scans. Wintergerst et al., using 3 × 3 mm swept-source OCTA scans, compared the retinal and choriocapillaris perfusion between patients with PAD (*n* = 52; mean age, 67.94 ± 9.47 years) and healthy controls (*n* = 23; mean age, 69.26 ± 10.31 years) and found that patients with PAD had a significant reduction in the retinal and choroidal circulation compared to age-matched healthy controls.^32^

## Discussion

Despite a high prevalence, PAD is underdiagnosed and untreated because the current recognition of PAD is suboptimal.^33^ Lack of an effective method to screen the population for PAD contributes to suboptimal physician detection and treatment of the condition. Currently, effective therapies that improve morbidity and mortality are available for individuals with PAD. Having an accessible and noninvasive novel biomarker can help refine risk stratification, determine prognosis, and determine the response to therapy. The focus of this review was to study the utility of ocular imaging as a biomarker for PAD diagnosis. The role of ocular imaging in the diagnosis of PAD has not been studied extensively. Systemic disease such as diabetes, hypertension, cardiovascular disease, and PAD often result in alterations in the circulation of both large and small vessels in peripheral organs, including the eye.^34^ The microvasculature of the eye consists of arteries, arterioles, venules, and capillaries and has high circulatory resistance making them highly susceptible. The pathogenesis of retinopathy seen in these underlying systemic disorders is generally attributed to chronic hypoxia because the retina has a higher oxygen demand than the brain.^35^ This leads to secondary changes in the blood vessels causing increa-sed retinal vascular tortuosity, retinal vascular dilatation, increased vascular branching, and a reduction in the RNFL thickness.^36^ Retinal imaging can assist in the early detection of these changes in patients with underlying systemic disease.

Our review included only five studies that used retinal imaging in PAD. These studies varied in terms of their study design, sample size, population, and the type of retinal imaging used. The OCT imaging technique was used by only one study and OCTA and color fundus photography were used in two studies each.

Of the two studies that used color fundus photography, one study used a wide-field fundus imaging system with a retinal view of 120°, whereas the other study used a nonmydriatic fundus imaging system with a retinal view of only 45°. In the first study, the authors used a multiple instance learning (MIL) algorithm to compare the retinal microvasculature between patients with PAD and controls, and demonstrated that alterations around the optic disc and temporal retinal vascular arcade were used to differentiate PAD from controls. However, in the second study, the authors used measures of retinopathy to predict PAD and reported that retinal hemorrhages, hard exudates, and microaneurysms were strongly associated with the development of PAD, especially the more severe form of the disease.

The study that used OCT to compare the retinal thickness, choroidal thickness, retinal artery diameter, and retinal vein diameter demonstrated that RNFL thickness in the nasal quadrant and the choroidal thickness in the subfoveal region were significantly reduced in patients with PAD compared to healthy individuals.

Two studies used the OCTA to assess retinal and choroidal perfusion in patients with PAD and controls; results showed that in patients with PAD both retinal and choroidal perfusions were reduced. PAD is a manifestation of the systemic disease atherosclerosis and the retinal vascular changes in PAD may be attributed to the systemic atherosclerotic burden. Although the reduced retinal and choroidal perfusion seen in OCTA in patients with PAD could be due to the atherosclerosis-associated reduction of the ocular blood flow, the RNFL thinning could be a secondary effect of the reduced retinal blood flow.^28^ Although all the studies demonstrated retinal microvascular changes in PAD, none of them investigated the association between the retinal changes and the disease severity or monitored the therapeutic response to medications/revascularization procedures. More studies investigating the utility of retinal imaging as a biomarker in PAD are needed in the future.

Whereas retinal blood flow exhibits autoregulation due to a high level of oxygen extraction and low blood flow, choroidal blood flow does not because of a low level of oxygen extraction and high level of blood flow.^37^ However, some studies have reported that choroid exhibits varying degrees of autoregulation.^38–40^ The blood flow velocities of the ophthalmic artery and central retinal artery are significantly reduced in patients with PAD compared to healthy controls.^28^ This impairment of blood flow velocity in the ophthalmic artery and central retinal artery may account for the retinal structural and blood flow changes seen in retinal imaging in patients with PAD.

Several prediction models have been developed for PAD including different potential variables such as age, sex, race, body mass index, pulse pressure, fasting glucose, total cholesterol (TC), TC/high-density lipoprotein (HDL) ratio, smoking, diabetes, hypertension, coronary artery disease and cerebrovascular disease, nonoral contraceptive pill usage, and parity.^41–44^ Smoking was substantially related to PAD in certain models, whereas the TC/HDL ratio was strongly associated with PAD in other models.^7,45^ Retinal imaging could aid as a quick, noninvasive, and inexpensive imaging biomarker in PAD. Incorporating the retinal parameters obtained from retinal imaging into the existing PAD risk assessment tools can enhance the risk stratification and guide response to treatment. Improvements in eye health reflect improvement in the systemic atherosclerotic burden and may, therefore, be key to determining optimal medication regimens for individuals. OCT/OCTA are very cost-effective and feasible, and are increasingly being used to diagnose and monitor systemic diseases such as diabetes, hypertension, Parkinson’s disease, and Alzheimer’s disease.^24,46^

### Study limitations

There were some limitations in the study. This review only included five studies that evaluated the utility of retinal imaging in patients with PAD. However, there is no minimum number of studies for inclusion in a systematic review and the number of studies that are included largely depends on the research topic as well as the amount of supportive evidence available. A smaller number of studies indicates a lack of research and knowledge gaps in this field. Moreover, all the studies that were included in this review were assessed for risk of bias using the risk of bias assessment tool. Another limitation was that only two studies used OCTA as an imaging modality to determine retinal changes in PAD. This could be because OCTA is a relatively new technique and its utility in a systemic disease such as PAD has not been well established. Another limitation was that none of the studies investigated the association between retinal changes and the severity of the disease. This might be because of the small sample size in these studies and future research examining the utility of retinal imaging in PAD should also investigate the relationship between retinal changes and the severity of the condition.

## Conclusion

To conclude, only a few studies have investigated the association between retinal microvascular changes (using OCT or OCTA imaging) and PAD. There is a strong clinical need for more specific biomarkers for PAD because clinical assessment for PAD has a relatively poor predictive value. Using a noninvasive ophthalmic imaging technique in PAD would significantly improve the rate of diagnosis without putting the patients at risk of complications. Early detection of PAD and subsequent treatment may have the potential to enhance quality of life and improve clinical outcomes for patients with PAD.

## Supplemental Material

sj-docx-1-vmj-10.1177_1358863X231210866 – Supplemental material for Role of noninvasive ocular imaging as a biomarker in peripheral artery disease (PAD): A systematic review Supplemental material, sj-docx-1-vmj-10.1177_1358863X231210866 for Role of noninvasive ocular imaging as a biomarker in peripheral artery disease (PAD): A systematic review by Mallika Prem Senthil, Chroran Kurban, Ngoc Thuy Nguyen, Anh-Phuong Nguyen, Ranjay Chakraborty, Christopher Delaney, Robyn Clark, Saumya Anand and Heena Bhardwaj in Vascular Medicine
