# Supplementary material for: Role of noninvasive ocular imaging as a biomarker in peripheral artery disease (PAD): A systematic review
Source: Vasc Med. 2023 Dec 6;29(2):215–22. doi: 10.1177/1358863X231210866 (PMC11010562; doi:10.1177/1358863X231210866)
Supplement: sj-docx-1-vmj-10.1177_1358863X231210866 – Supplemental material for Role of noninvasive ocular imaging as a biomarker in peripheral artery disease (PAD): A systematic review [file sj-docx-1-vmj-10.1177_1358863X231210866.docx]

Supplementary material 1. Databases and search terms used for the systematic review.

**Database_ Medline**

| Ovid MEDLINE(R) and Epub Ahead of Print, In-Process, In-Data-Review & Other Non-Indexed Citations, Daily and Versions <1946 to November 11, 2022> | | |
| --- | --- | --- |
| 1 | peripheral vascular diseases/ or exp peripheral arterial disease/ | 23345 |
| 2 | ((Peripheral vascular or Peripheral arter* or lower extremity arterial*) adj3 disease*).tw,kf. | 31729 |
| 3 | (peripheral adj3 (arteriopath* or angiopath*)).tw,kf. | 588 |
| 4 | ("Vascular disease" or "Hardening of the arteries").tw,kf. | 36440 |
| 5 | ((poor or bad) adj2 circulation).tw,kf. | 516 |
| 6 | (Claudication or PAOD or PAD or dysvascular*).tw,kf. | 40154 |
| 7 | ((diabetic or diabetes) adj3 foot).tw,kf. | 11883 |
| 8 | ((leg or limb or peripheral or arm or foot or feet or hand or lower extremity) adj4 (obstruction or occlusion or gangrene or ischemia)).tw,kf. | 18198 |
| 9 | or/1-8 | 120964 |
| 10 | optical imaging/ or narrow band imaging/ or tomography, optical/ or tomography, optical coherence/ or transillumination/ or Tomography, Optical Coherence/ | 63420 |
| 11 | diagnostic imaging/ or exp image interpretation, computer-assisted/ or exp imaging, three-dimensional/ or tomography/ or Computed Tomography Angiography/ | 696929 |
| 12 | (transillumination or tomograph*).tw,kf. | 505929 |
| 13 | (imag* adj3 (computer* or three-dimension* or 3D or 3-d or interpret* or diagnos* or fluorescence)).tw,kf. | 142534 |
| 14 | or/10-13 | 1057709 |
| 15 | exp Retina/ | 149905 |
| 16 | (retina* or fundus or choroid or macular or fovea or amacrine or photorecptor*).tw,kf. | 279310 |
| 17 | or/15-16 | 315418 |
| 18 | 9 and 14 and 17 | 278 |
| 19 | exp animals/ not humans.sh. | 5063641 |
| 20 | 18 not 19 | 269 |

**2. Database_Embase**

| Embase <1974 to 2022 November 11> | |  |
| --- | --- | --- |
| 1 | peripheral vascular disease/ or exp artery occlusion/ or artery disease/ or peripheral vascular disease/ or exp arterial insufficiency/ or exp arterial wall thickening/ or exp arteriosclerosis/ or exp artery calcification/ or exp peripheral occlusive artery disease/ | 474737 |
| 2 | ((Peripheral vascular or Peripheral arter* or lower extremity arteral*) adj3 disease*).tw,kw. | 45509 |
| 3 | (peripheral adj3 (arteriopath* or angiopath*)).tw,kw. | 623 |
| 4 | ("Vascular disease" or "Hardening of the arteries").tw,kw. | 52750 |
| 5 | ((poor or bad) adj2 circulation).tw,kw. | 731 |
| 6 | (Claudication or PAOD or PAD or dysvascular*).tw,kw. | 59146 |
| 7 | ((diabetic or diabetes) adj3 foot).tw,kw. | 16702 |
| 8 | ((leg or limb or peripheral or arm or foot or feet or hand or lower extremity) adj4 (obstruction or occlusion or gangrene or ischemia)).tw,kw. | 25985 |
| 9 | or/1-8 | 574481 |
| 10 | fluorescence imaging/ or narrow band imaging/ or optical tomography/ or optical coherence tomography/ or transillumination/ | 108560 |
| 11 | computer assisted tomography/ or four dimensional computed tomography/ or high resolution computer tomography/ or computed tomographic angiography/ or diagnostic imaging/ | 1124714 |
| 12 | (transillumination or tomograph*).tw,kw. | 623083 |
| 13 | (imag* adj3 (computer* or three-dimension* or 3D or 3-d or interpret* or diagnos* or fluorescence)).tw,kw. | 186543 |
| 14 | or/10-13 | 1561077 |
| 15 | exp retina/ | 148089 |
| 16 | (retina* or fundus or choroid or macular or fovea or amacrine or photorecptor*).tw,kw. | 341632 |
| 17 | or/15-16 | 362682 |
| 18 | 9 and 14 and 17 | 1723 |
| 19 | (exp animal/ or exp invertebrate/ or nonhuman/ or animal experiment/ or animal tissue/ or animal model/ or exp plant/ or exp fungus/) not (exp human/ or human tissue/) | 7486937 |
| 20 | 18 not 19 | 1666 |
| 21 | limit 20 to (embase and (article or article in press) and journal) | 905 |

3. **Database_Scopus**

( TITLE-ABS-KEY ( ( retina* OR fundus OR choroid OR macular OR fovea OR amacrine OR photorecptor* ) ) ) AND ( TITLE-ABS-KEY ( ( transillumination OR tomograph* ) OR ( imag* W/3 ( computer* OR three-dimension* OR 3d OR 3-d OR interpret* OR diagnos* OR fluorescence ) ) ) ) AND ( ( TITLE-ABS-KEY ( ( ( "Peripheral vascular" OR "Peripheral arter*" OR "lower extremity arteral*" ) W/3 disease* ) OR ( "Vascular disease" OR "Hardening of the arteries" ) OR ( ( poor OR bad ) W/2 circulation ) OR ( peripheral W/3 ( arteriopath* OR angiopath* ) ) OR ( claudication OR paod OR pad OR dysvascular* ) ) OR TITLE-ABS-KEY ( ( ( diabetic OR diabetes ) W/3 foot ) OR ( ( leg OR limb OR peripheral OR arm OR foot OR feet OR hand OR "lower extremity" ) W/4 ( obstruction OR occlusion OR gangrene OR ischemia ) ) ) ) ) AND ( LIMIT-TO ( DOCTYPE, "ar" ) ) AND ( LIMIT-TO ( LANGUAGE, "English" ) )

**4. Database_LILACS**

((("Peripheral vascular" OR "Peripheral arter*" OR "lower extremity arteral*") AND disease*) OR (peripheral AND (arteriopath* OR angiopath*)) OR ("Vascular disease" OR "Hardening of the arteries") OR ((poor OR bad) AND circulation) OR (Claudication OR PAOD OR PAD OR dysvascular*) OR ((diabetic OR diabetes) AND foot) OR ((leg OR limb OR peripheral OR arm OR foot OR feet OR hand OR "lower extremity") AND (obstruction OR occlusion OR gangrene OR ischemia))) AND (retina* OR fundus OR choroid OR macular OR fovea OR amacrine OR photorecptor*) AND (transillumination OR tomograph* OR (imag* AND (computer* OR three-dimension* OR 3D OR "3-d" OR interpret* OR diagnos* OR fluorescence)))

**5. Database_Cochrane**

(((("Peripheral vascular" OR "Peripheral arter*" OR "lower extremity arteral*") NEAR/3 disease*) OR (peripheral NEAR/3 (arteriopath* OR angiopath*)) OR ("Vascular disease" OR "Hardening of the arteries") OR ((poor OR bad) NEAR/2 circulation) OR (Claudication OR PAOD OR PAD OR dysvascular*) OR ((diabetic OR diabetes) NEAR/3 foot) OR ((leg OR limb OR peripheral OR arm OR foot OR feet OR hand OR "lower extremity") NEAR/4 (obstruction OR occlusion OR gangrene OR ischemia))) AND (retina* OR fundus OR choroid OR macular OR fovea OR amacrine OR photorecptor*) AND (transillumination OR tomograph* OR (imag* NEAR/3 (computer* OR three-dimension* OR 3D OR "3-d" OR interpret* OR diagnos* OR fluorescence)))):ti,ab,kw

**6. Database_Master search**

((("Peripheral vascular" OR "Peripheral arter*" OR "lower extremity arteral*") NEAR/3 disease*) OR (peripheral NEAR/3 (arteriopath* OR angiopath*)) OR ("Vascular disease" OR "Hardening of the arteries") OR ((poor OR bad) NEAR/2 circulation) OR (Claudication OR PAOD OR PAD OR dysvascular*) OR ((diabetic OR diabetes) NEAR/3 foot) OR ((leg OR limb OR peripheral OR arm OR foot OR feet OR hand OR "lower extremity") NEAR/4 (obstruction OR occlusion OR gangrene OR ischemia)))

AND

(retina* OR fundus OR choroid OR macular OR fovea OR amacrine OR photorecptor*)

AND

(transillumination OR tomograph* OR (imag* NEAR/3 (computer* OR three-dimension* OR 3D OR "3-d" OR interpret* OR diagnos* OR fluorescence)))

**Supplementary material 2. Assessment of risk of bias of the studies included in the systematic review using the National Institute of Health Study Quality Assessment Tools for case-control studies.**

| **Criteria** | **Mueller *et al*** | **Soydan *et al*** | **Wintergest *et al*** |
| --- | --- | --- | --- |
| Was the research question or objective in this paper clearly stated and appropriate? | Yes | Yes | Yes |
| Was the study population clearly specified and defined? | Yes | Yes | Yes |
| Did the authors include a sample size justification? | No | No | No |
| Were controls selected or recruited from the same or similar population that gave rise to the cases (including the same timeframe)? | Yes | Yes | Yes |
| Were the definitions, inclusion and exclusion criteria, algorithms or processes used to identify or select cases and controls valid, reliable, and implemented consistently across all study participants? | Yes | Yes | Yes |
| Were the cases clearly defined and differentiated from controls? | Yes | Yes | Yes |
| If less than 100 percent of eligible cases and/or controls were selected for the study, were the cases and/or controls randomly selected from those eligible? | Not applicable | Not applicable | Not applicable |
| Was there use of concurrent controls? | Not reported | Not reported | Not reported |
| Were the investigators able to confirm that the exposure/risk occurred prior to the development of the condition or event that defined a participant as a case? | Yes | Yes | Yes |
| Were the measures of exposure/risk clearly defined, valid, reliable, and implemented consistently (including the same time period) across all study participants? | Yes | Yes | Yes |
| Were the assessors of exposure/risk blinded to the case or control status of participants? | Not reported | Not reported | Not reported |
| Were key potential confounding variables measured and adjusted statistically in the analyses? If matching was used, did the investigators account for matching during study analysis? | Not applicable | Not reported | Yes |

**Supplementary material 2. Assessment of risk of bias of the studies included in the systematic review using the National Institute of Health Study Quality Assessment Tools for Observational cohort and cross-sectional studies.**

| **Criteria** | **Yang *et al*** | **Sun *et al*** |
| --- | --- | --- |
| Was the research question or objective in this paper clearly stated? | Yes | Yes |
| Was the study population clearly specified and defined? | Yes | Yes |
| Was the participation rate of eligible persons at least 50%? | Yes | Yes |
| Were all the subjects selected or recruited from the same or similar populations (including the same time period)? Were inclusion and exclusion criteria for being in the study prespecified and applied uniformly to all participants? | Yes | Yes |
| Was a sample size justification, power description, or variance and effect estimates provided? | No | No |
| For the analyses in this paper, were the exposure(s) of interest measured prior to the outcome(s) being measured? | Yes | Not applicable |
| Was the timeframe sufficient so that one could reasonably expect to see an association between exposure and outcome if it existed? | Yes | Not applicable |
| For exposures that can vary in amount or level, did the study examine different levels of the exposure as related to the outcome (e.g., categories of exposure, or exposure measured as continuous variable)? | Yes | Not applicable |
| Were the exposure measures (independent variables) clearly defined, valid, reliable, and implemented consistently across all study participants? | Yes | Yes |
| Was the exposure(s) assessed more than once over time? | Yes | No |
| Were the outcome measures (dependent variables) clearly defined, valid, reliable, and implemented consistently across all study participants? | Yes | Yes |
| Were the outcome assessors blinded to the exposure status of participants? | Not reported | No |
| Was loss to follow-up after baseline 20% or less? | Not reported | Not applicable |
| Were key potential confounding variables measured and adjusted statistically for their impact on the relationship between exposure(s) and outcome(s)? | Yes | Yes |
